# Study on the optimal elastic modulus of flexible blades for right heart assist device supporting patients with single-ventricle physiologies

**DOI:** 10.3389/fcvm.2024.1377765

**Published:** 2024-03-25

**Authors:** Tong Chen, Xiaoming Cheng, Xudong Liu, Huifeng Zhang, Shengzhang Wang

**Affiliations:** ^1^ Academy for Engineering and Technology, Fudan University, Shanghai, China; ^2^ Department of Aeronautics and Astronautics, Fudan University, Shanghai, China; ^3^ Shanghai Key Laboratory of Interventional Medical Devices and Equipment, Shanghai MicroPort Medical Group Co., Ltd, Shanghai, China; ^4^ Department of Cardiothoracic Surgery, Children’s Hospital of Fudan University, Shanghai, China

**Keywords:** single-ventricle physiology, right heart assist device, flexible blade, one-way fluid-structure interaction simulation, elastic modulus

## Abstract

**Background:**

Patients with single-ventricle physiologies continue to experience insufficient circulatory power after undergoing palliative surgeries. This paper proposed a right heart assist device equipped with flexible blades to provide circulatory assistance for these patients. The optimal elastic modulus of the flexible blades was investigated through numerical simulation.

**Methods:**

A one-way fluid-structure interaction (FSI) simulation was employed to study the deformation of flexible blades during rotation and its impact on device performance. The process began with a computational fluid dynamics (CFD) simulation to calculate the blood pressure rise and the pressure on the blades’ surface. Subsequently, these pressure data were exported for finite element analysis (FEA) to compute the deformation of the blades. The fluid domain was then recreated based on the deformed blades’ shape. Iterative CFD and FEA simulations were performed until both the blood pressure rise and the blades’ shape stabilized. The blood pressure rise, hemolysis risk, and thrombosis risk corresponding to blades with different elastic moduli were exhaustively evaluated to determine the optimal elastic modulus.

**Results:**

Except for the case at 8,000 rpm with a blade elastic modulus of 40 MPa, the pressure rise associated with flexible blades within the studied range (rotational speeds of 4,000 rpm and 8,000 rpm, elastic modulus between 10 MPa and 200 MPa) was lower than that of rigid blades. It was observed that the pressure rise corresponding to flexible blades increased as the elastic modulus increased. Additionally, no significant difference was found in the hemolysis risk and thrombus risk between flexible blades of various elastic moduli and rigid blades.

**Conclusion:**

Except for one specific case, deformation of the flexible blades within the studied range led to a decrease in the impeller’s functionality. Notably, rotational speed had a more significant impact on hemolysis risk and thrombus risk compared to blade deformation. After a comprehensive analysis of blade compressibility, blood pressure rise, hemolysis risk, and thrombus risk, the optimal elastic modulus for the flexible blades was determined to be between 40 MPa and 50 MPa.

## 1 Introduction

Congenital heart defects are the most prevalent type of birth defect, occurring in approximately 9 out of every 1,000 live births (1). Among these, 9%–12% are classified as single ventricle physiologies (2). Patients with single-ventricle physiologies often have atrial and ventricular septal defects, leading to the mixing of arterial and venous blood within the heart chambers. A portion of the venous blood enters the systemic circulation without being oxygenated, causing organ hypoxia. Typically, patients undergo three-stage palliative surgeries: the Norwood, Glenn, and Fontan procedures (3). After the Fontan procedure, patient's superior and inferior vena cava (SVC and IVC, respectively) are connected to the pulmonary artery (PA), forming a total cavopulmonary connection (TCPC) structure. This setup forces the single ventricle to pump blood through both systemic and pulmonary circulations in the absence of a functional right ventricle. Over time, this can result in high central venous pressure and low pulmonary artery pressure (known as Fontan failure) (4). Approximately 40% of patients eventually suffer from single ventricle pumping failure (5).

Assistive devices play a critical role in augmenting circulatory power for these patients. In 2010, Throckmorton et al. (6) introduced an axial blood pump into the IVC to aid blood flow to the lungs. However, as the outlet of the blood pump is oriented towards the SVC, the blood flow pressurized by the pump collides with the blood flow within the SVC. This collision leads to an increase in blood pressure inside the SVC and hinders blood return. Then in 2013, Throckmorton et al. (7) implanted axial blood pumps in both SVC and IVC to provide dual-lumen assistance. In 2014, Wang et al. (8) used a paired umbrella double-lumen cannula (Maquet Cardiovascular, Wayne, NJ) and extracorporeal blood pump CentriMag (Thoratec, Pleasanton, CA) for total cavopulmonary assist. Animal studies revealed that this setup effectively improved hemodynamics in failing Fontan sheep. Subsequently, in 2019, Wang et al. (9) combined a double-lumen cannula with a two-valve extracardiac conduit and CentriMag to achieve effective support. In 2016, Gandolfo et al. (10) designed a T-shaped mechanically assisted TCPC model using the Jarvik Child 2,000 axial pump (Jarvik Heart, New York, NY, USA), which showed promising results *in vivo*. Although these techniques can provide total cavopulmonary assist, they have drawbacks such as the need for multiple devices, limitations on patient activity due to extracorporeal blood pumps, and the necessity for vascular reconfiguration.

**Figure 1:**
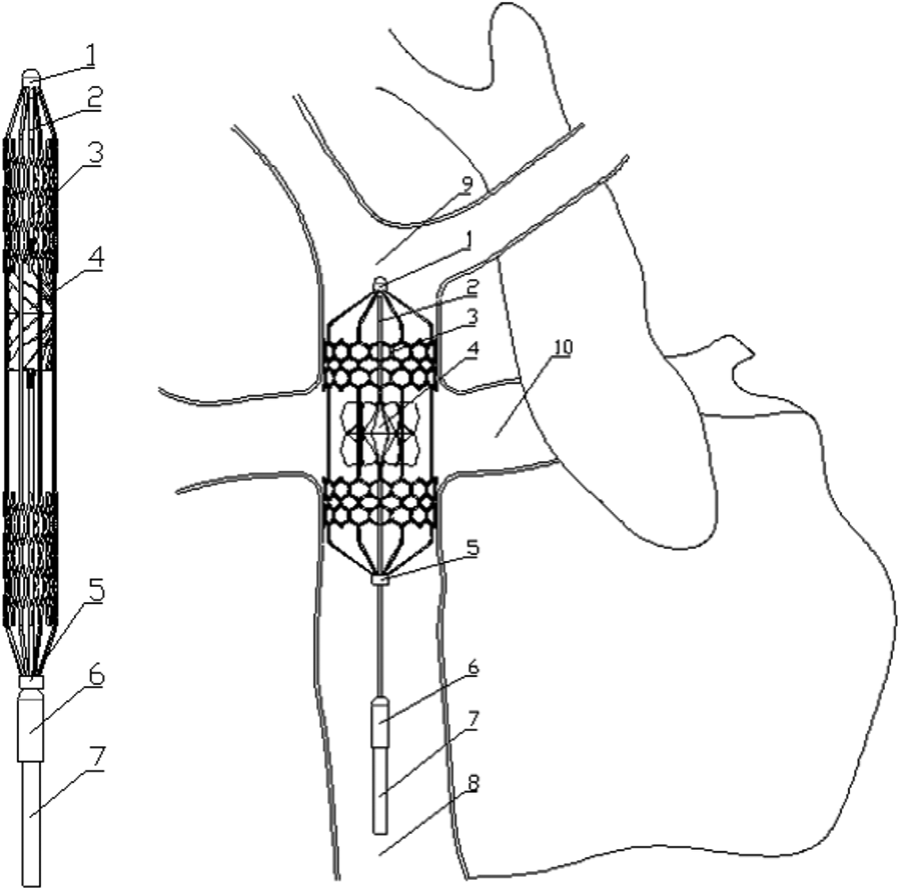
Contraction state and post-implantation state of the flexible right heart assist device. (1. bearing seat; 2. shaft; 3. protective stent; 4. impeller; 5. constrictor ring; 6. motor; 7. cable; 8. inferior vena cava; 9. superior vena cava; 10. pulmonary artery).

To address the issues mentioned above, this study proposed a novel right heart assist device, as shown in Figure 1. The main components of this device are made of flexible materials. During minimally invasive surgery, the device is in a contracted state with a smaller diameter. Once implanted in the human body, the device expands and begins to function. It is anticipated that the deformation of flexible components during operation will impact the device's performance. Consequently, ensuring rigidity in its expanded state while maintaining flexibility during folding poses the principal challenge in developing this device. The material selection for each component is crucial. This research focuses on a key component—the blades, investigating their optimal elastic modulus. A one-way FSI simulation was employed to study blades’ deformation during rotation, and their impact on blood pressure increase, hemolysis risk, and thrombosis risk. The optimal elastic modulus is determined through a comprehensive analysis of these indicators.

## 2 Materials and methods

### 2.1 Flexible right heart assist device and computational models

Figure 1 shows the proposed right heart assist device, consisting of a protective stent, a bearing seat, a bearing, a shaft, an impeller, a constrictor ring, a motor, and cables. The protective stent is made of Nitinol. One end of the stent is fixed to the bearing seat, while the other end connects to the constrictor ring and can move axially. The impeller is fabricated from flexible materials such as polyurethane through an integrated molding process. It consists of two parts: the hub and the blades, with the base of the blades affixed to the surface of the hub. The hub's upper end is mounted on the shaft, and its lower end can move axially. The blades can adopt two configurations: curled and unfolded. When the assist device contracts, the lower end of the hub moves downward, and the blades curl. As the device expands, both the hub and the blades revert to their initial shape. The motor is powered by cables, and drives the rotation of the shaft and impeller. During surgery, the entire device is compressed within a delivery catheter and implanted into the TCPC via the femoral vein. Once released, the protective stent expands to tightly fit against the inner walls of the vena cava, stabilizing the device. The rotating impeller aids in directing blood within the SVC and IVC into the PA. Additionally, the hub is uniquely designed in a spindle shape to prevent blood from colliding. The design goal of this assist device is to provide a pressure rise of 2–25 mmHg for the total systemic blood flow (3l /min 11) in Fontan patients when operating at 4,000–8,000 rpm (12).

**Figure 2:**
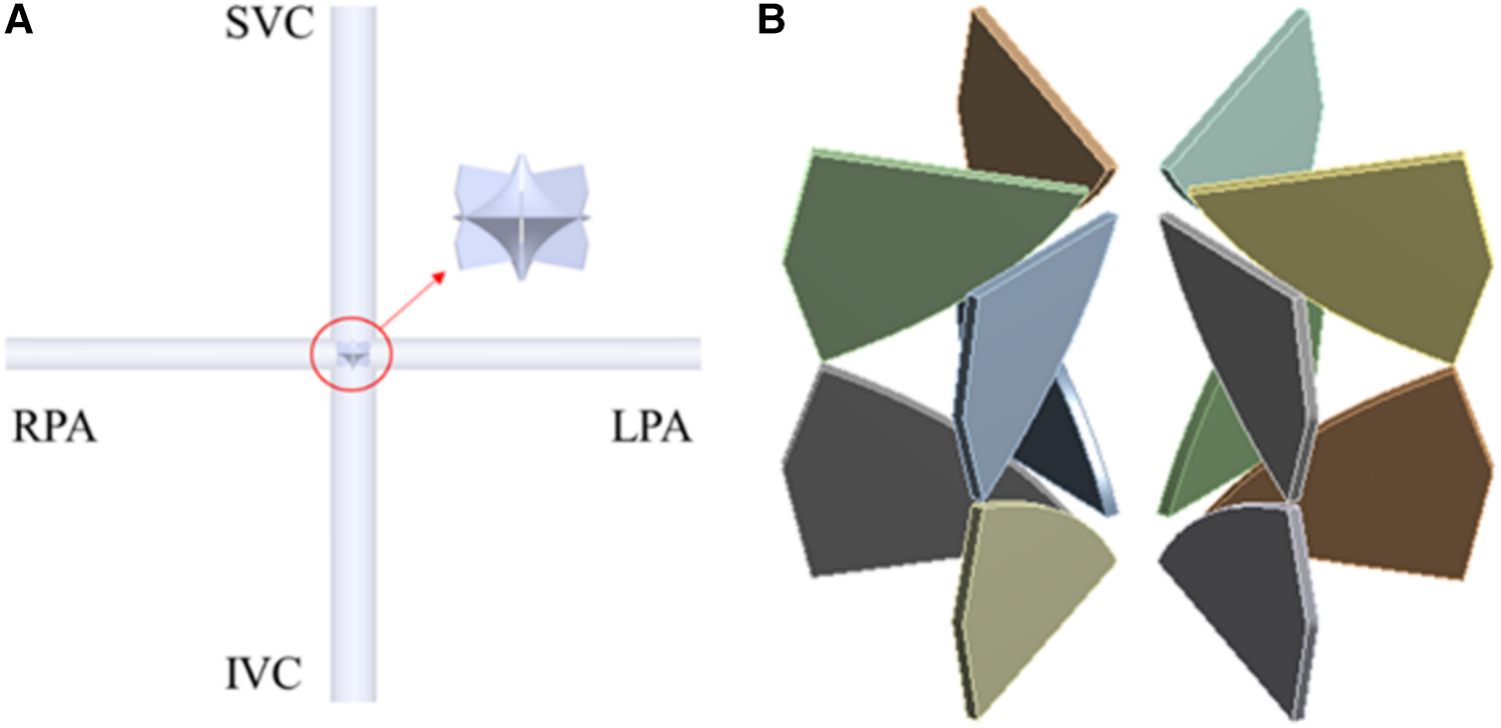
CFD and FEA simulation model. (**A**) Ideal TCPC structure; (**B**) blades of right heart assist device.

This paper focuses on flexible blades, therefore adopts simplified computational models. The TCPC model is an ideal circular tube model derived from patient's data (13). According to the principle of equal area, the diameters of the SVC and IVC are set at 13 mm, while those of the LPA and RPA are set at 9 mm. In CFD simulation, only the impeller is retained. The base of the blades is fixed to the hub's surface to ensure the model's consistency with reality. The minimum and maximum diameters of the impeller are 0.4 mm and 10.9 mm respectively, the number of blades is 12, and the thickness of the blades is 0.2 mm. In FEA simulation, only the blades are retained. The deformation of the hub is ignored. The impeller and blade models presented in this paper are the optimized versions after structural optimization. Figure 2 illustrates the final computational models used in this study.

### 2.2 One-way FSI methodology, boundary conditions and solving settings

**Figure 3:**
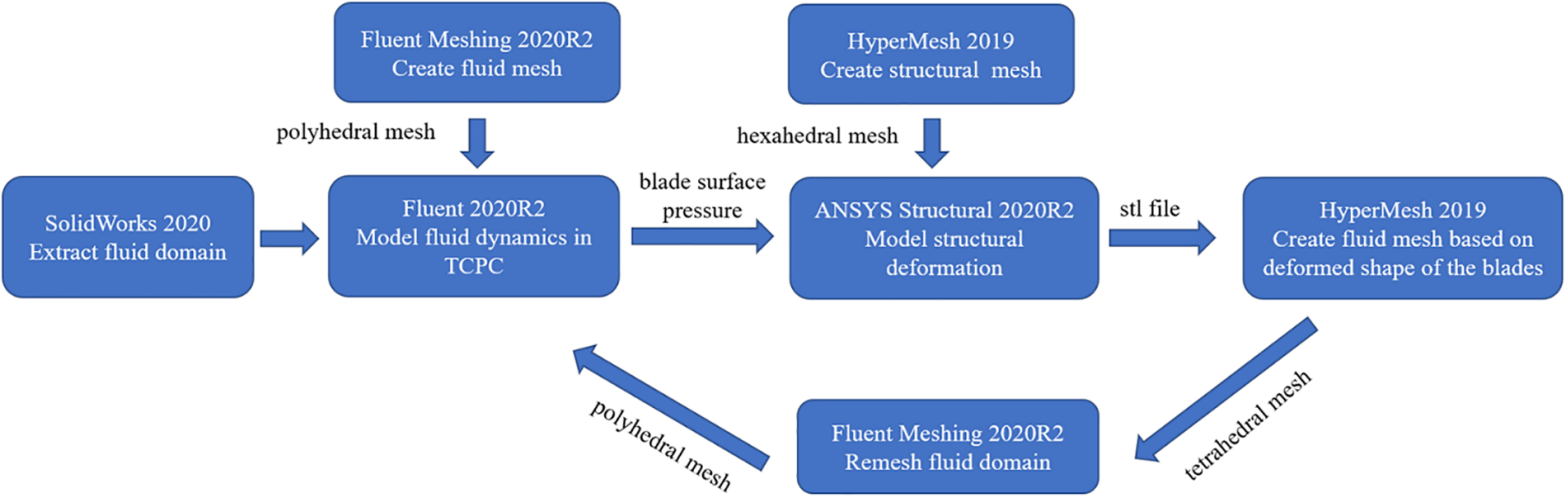
One-way FSI simulation process.

In this study, one-way FSI simulation was used to analyze the flexible blades’ deformation and its effect on the device performance, as shown in Figure 3. First, ANSYS Fluent 2020R2 (Canonsburg, USA) was used to model the fluid dynamics in TCPC and obtain the blade surface pressure. Subsequently, these pressure data were utilized in ANSYS Structural 2020R2 (Canonsburg, USA) to compute blades’ deformation. The deformation of the blades was quantitatively described by the displacement of nodes on the blades. After the deformed shape of the blades was exported, the fluid domain was remeshed. Subsequently, the CFD simulation was performed again. The cycle was ended when the difference in blood pressure rise between two consecutive CFD simulations was less than 5%. Finally, the last CFD result was used to calculate the hemolysis risk and thrombus risk.

In CFD simulation, flow boundary conditions were defined at the SVC and IVC inlets, with flow rates of 1.2l /min and 1.8l /min respectively (11). The LPA and RPA outlets were set as pressure boundary conditions, with a pressure value of 1,750 Pa, corresponding to the average pulmonary artery pressure during a patient's cardiac cycle (13). Blood was modeled as a Newtonian fluid with a density of 1,060 kg/m^3^ and a dynamic viscosity of 3.5 cP. This article analyzes the operation of the assist device at its lowest and highest rotational speeds. The flow domain is divided into rotating and stationary regions. The rotational speed of the rotating region is set at 4,000 rpm and 8,000 rpm. Furthermore, rotational boundary conditions are applied to the surfaces of the hub and blades, with the rotational speed settings matching those of the rotating region. The remaining surfaces are treated as stationary boundaries. Additionally, the SST k-*ω* model was used to simulate turbulent conditions, as this model can accurately resolve the flow fields both at the wall and in the bulk flow regime (12, 14, 15). The fluid domain was meshed as a polyhedral grid using Fluent Meshing 2020R2 (Canonsburg, USA). After grid independence study, the fluid mesh consisting of approximately 1.35 million elements was used for CFD simulation. Inflation layers were also used in the fluid meshes to achieve the SST k-*ω* turbulence model requirement (y + less than 2). A steady-state solver was employed, with the convergence criteria was set to a maximum residual of below the 1 × 10^−4^ threshold.

**Figure 4:**
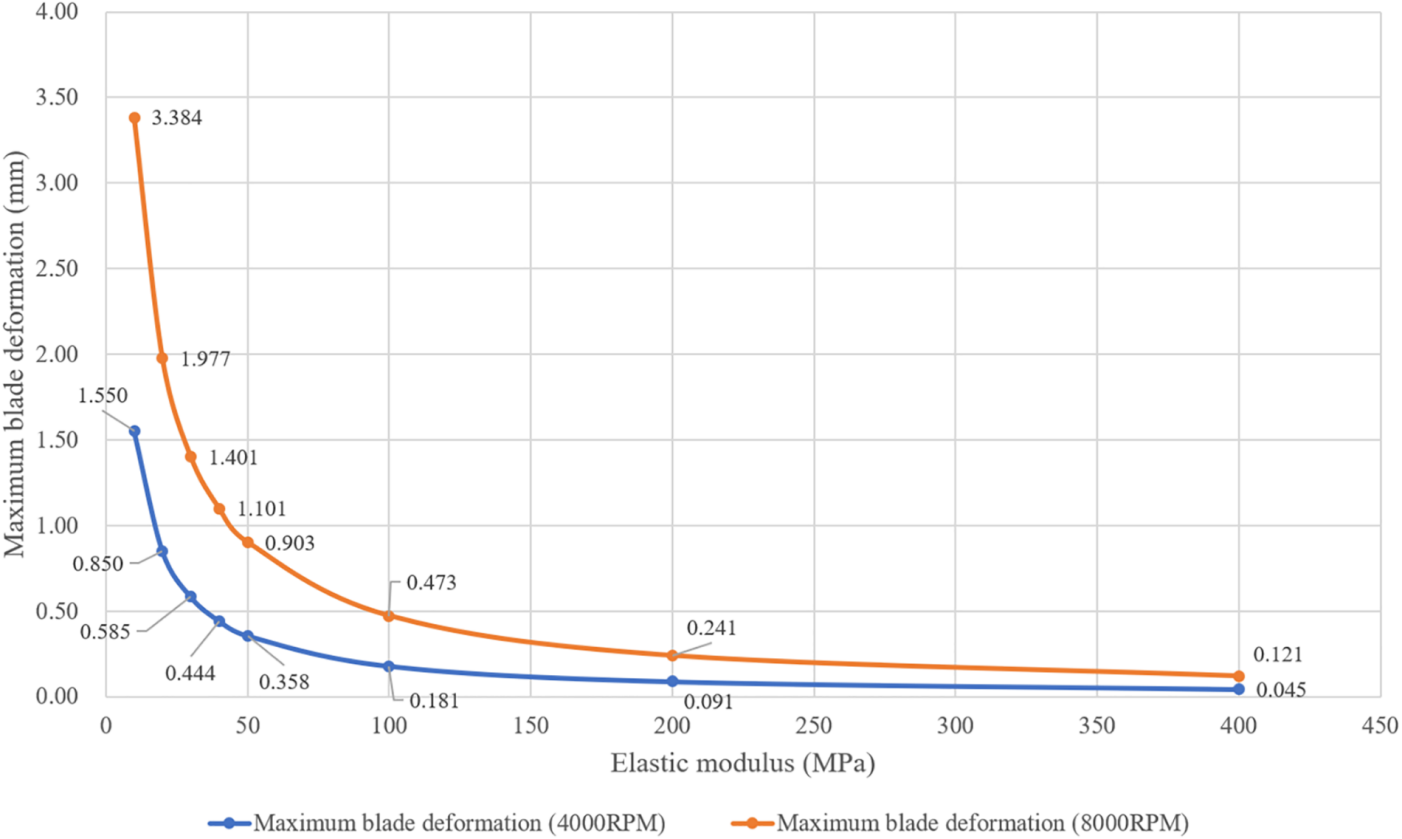
Maximum deformation of blades with different elastic moduli at rotational speeds of 4,000 rpm and 8,000 rpm. (These results were obtained from one round of one-way FSI simulation).

In FEA simulation, the blade material was set based on the polyurethane material properties. There is a wide variety of linear elastic polyurethanes, with elastic moduli ranging from 6.2 MPa to 27,600 MPa (16). A pre-simulation (a single round of one-way FSI) was conducted to narrow down the range of elastic moduli for the study, as shown in Figure 4. At 4,000 rpm and 8,000 rpm, the maximum deformation of the blades showed a consistent pattern. When the elastic modulus is within 50 MPa, the maximum deformation decreases rapidly as the elastic modulus increases. Between 50 MPa and 200 MPa, the decrease in maximum deformation is more gradual. Above 200 MPa, both the absolute value and the variation in maximum deformation are extremely small. Based on these results, the elastic moduli selected for the study were 10 MPa, 20 MPa, 30 MPa, 40 MPa, 50 MPa, 100 MPa, and 200 MPa. In the actual configuration, the base of the blades is fixed to the hub's surface, leading to the application of a fixed constraint at the base of the blades. The structural mesh of the blade, generated using HyperMesh (Altair, USA), is a hexahedral mesh and consists of two layers along the thickness direction. After the grid independence study, the structural mesh consisting of 36,096 elements was used for FEA simulation. A transient solver was employed, with a total computational duration of 1.1 s and a time step of 0.001 s. The convergence criteria was set as changes of the maximum deformation, the minimum deformation, and the average deformation are all less than 1%.

### 2.3 Hemolysis risk assessment model

During the high-speed rotation of the impeller, red blood cells are destroyed due to shear forces, resulting in hemolysis. The hemolysis risk can be quantitatively evaluated using the Normalized index of hemolysis (NIH). Numerical simulations and hemolysis experiments employ different calculation methods to derive this index. In numerical simulation, NIH is commonly derived through a power law model, as detailed in Equations 1–3 (17–19).

(1) $$\sigma ={\left(\frac{1}{6}\sum {\left(\sigma_{\mathrm{ii}}-\sigma_{\mathrm{jj}}\right)}^{2}+\sum \sigma_{\mathrm{ij}}^{2}\right)}^{\frac{1}{2}}$$

(2) $$D=\sum_{\mathrm{inlet}}^{\mathrm{outlet}}C\cdot \sigma^{\alpha }\cdot \Delta T^{\beta }$$

(3) NIH=0.00015∗D

where *σ* is the scalar stress, ΔT is the exposure time, and C,α,β are constants obtained from hemolysis experiments. Mitamura et al. (20) derived a set of parameters based on the experimental data from axial blood pumps: $C=3.62\times 10^{-5},\;\alpha =2.416,\;\beta =0.785$. Heuser et al. (21) used experimental data from a Couette viscometer to fit another set of parameters: $C\;=1.8\times 10^{-6},\;\alpha =1.991,\;\beta =0.765$. The working principle of the impeller in this paper differs considerably from that of the axial blood pump and Couette viscometer, and is closer to that of the centrifugal blood pump. Therefore, this paper uses experimental data of centrifugal blood pumps, provided by the U.S. Food and Drug Administration (FDA), to determine the parameters of the hemolysis risk assessment model (22).

In addition, Blackshear et al. (23) conducted a hemolysis experiment and proposed the relationship between shear stress experienced by red blood cells and their exposure time:

(4) $$(\tau_{\mathrm{rbc}}^{2})(t)=C_{1}$$

In the above equation $C_{1}$ is a constant of proportionality. The results of Blackshear et al. indicate that the hemolysis risk is proportional to the exposure time and the square of the shear stress. Combined with Equations 1–4, the final hemolysis risk assessment model used in this paper is as follows:

(5) $$\sigma ={\left(\frac{1}{6}\sum {\left(\sigma_{\mathrm{ii}}-\sigma_{\mathrm{jj}}\right)}^{2}+\sum \sigma_{\mathrm{ij}}^{2}\right)}^{\frac{1}{2}}$$

(6) $$NIH=\sum_{\mathrm{inlet}}^{\mathrm{outlet}}A\cdot \sigma^{2B}\cdot \Delta T^{B}$$

In Equation 6, A represents the overall scaling factor, set at 2.7 × 10^−10^. This value was derived by multiplying the scaling factor C, provided by Heuser (21), with the scaling factor of 0.00015 from Equation 3. The parameter B, set at 0.55, was determined from experimental data, as detailed in the Supplementary Document.

### 2.4 Thrombus risk assessment model

Thrombus formation is another common complication associated with rotary assist devices (24). It involves the generation, transport, and cascade reactions of numerous components. The adhesion and activation of platelets are critical steps in thrombus formation. Cell adhesion only occurs in areas where the wall shear strain rate $\left(\dot{\gamma }\right)$ is lower than 100 /s (25). Therefore, the shear strain rate can be used to identify areas prone to thrombus growth (26). Its calculation method is shown in Equation 7. An accelerated thrombus formation model is utilized to quantitatively assess the thrombus risk (27). This model includes three convection-diffusion equations (Equations 8–10), describing the generation and transport of non-activated platelets (NP), activated platelets (AP), and adenosine diphosphate (ADP). The concentration of AP is ultimately used to represent the thrombus risk.

(7) $$\dot{\gamma }={\left[2\left({\left(\frac{\partial u}{\partial x}\right)}^{2}+{\left(\frac{\partial v}{\partial y}\right)}^{2}+{\left(\frac{\partial w}{\partial z}\right)}^{2}\right)+{\left(\frac{\partial u}{\partial y}+\frac{\partial v}{\partial x}\right)}^{2}+{\left(\frac{\partial u}{\partial z}+\frac{\partial w}{\partial x}\right)}^{2}+{\left(\frac{\partial v}{\partial z}+\frac{\partial w}{\partial y}\right)}^{2}\right]}^{\frac{1}{2}}$$

(8) $$\frac{\partial (\phi_{a})}{\partial t}+(u\cdot \nabla )\phi_{a}=D_{a}\nabla^{2}\phi_{a}+\{\left[A_{C}(ADP)]\phi_{n}+[A_{M}(\phi_{f},\tau )](\phi_{a}+\phi_{n}\right)\}$$

(9) $$\frac{\partial (\phi_{n})}{\partial t}+(u\cdot \nabla )\phi_{n}=D_{n}\nabla^{2}\phi_{n}-\{\left[A_{C}(ADP)]\phi_{n}+[A_{M}(\phi_{f},\tau )](\phi_{a}+\phi_{n}\right)\}$$

(10) $$\frac{\partial (ADP)}{\partial t}+(u\cdot \nabla )ADP=D_{ADP}\nabla^{2}ADP+R_{ADP}\{\left[A_{C}(ADP)]\phi_{n}+[A_{M}(\phi_{f},\tau )](\phi_{a}+\phi_{n}\right)\}$$

In the above equations, *D_x_* is the respective diffusivity coefficient, *A_M_* and *A_C_* denotes mechanical activation and chemical activation, and *R_ADP_* is the amount of ADP contained in a platelet. Platelet activation (change from NP to AP) occurs either with mechanical cues (Equation 11) or chemical cues (Equation 12).

(11) $$A_{M}(\phi_{f},\tau )=(1-\phi_{f})C_{1}^{\frac{1}{\beta_{1}}}\beta_{1}\phi_{f}^{\frac{\beta_{1}-1}{\beta_{1}}}\tau^{\frac{\alpha_{1}}{\beta_{1}}}$$

(12) $$A_{C}(\mathrm{ADP})=\{\begin{array}{c} \frac{\mathrm{ADP}}{\mathrm{AD}P_{t}\cdot t_{\mathrm{ADP}}},\mathrm{ADP}\geq \mathrm{AD}P_{t}\\ 0\;\;\;\;\;\;\;\;\;\;\;\;\;\;\;\;\;\;,\;\mathrm{ADP}<\mathrm{AD}P_{t} \end{array}$$

where $\phi_{f}$ is the ratio of the number of activated platelets to total platelets count, $\alpha_{1}$, $\beta_{1}$ and $C_{1}$ are the power law model parameter, $\mathrm{AD}P_{t}$ is the threshold for chemical platelet activation, $t_{\mathrm{ADP}}$ is the characteristic time of platelet activation, and *τ* is the shear stress calculated from Equations 13–15.

(13) $$\tau =\frac{1}{\sqrt{3}}\sqrt{\sigma_{\mathrm{xx}}^{2}+\sigma_{\mathrm{yy}}^{2}+\sigma_{\mathrm{zz}}^{2}-\sigma_{\mathrm{xx}}\sigma_{\mathrm{yy}}-\sigma_{\mathrm{yy}}\sigma_{\mathrm{zz}}-\sigma_{\mathrm{xx}}\sigma_{\mathrm{zz}}+3(\sigma_{\mathrm{xy}}^{2}+\sigma_{\mathrm{yz}}^{2}+\sigma_{\mathrm{xz}}^{2})}$$

(14) $$\sigma_{\mathrm{ij}}=\mu \left(\frac{\partial u_{i}}{\partial x_{j}}+\frac{\partial u_{j}}{\partial x_{i}}\right)-\rho \bar{u_{i}^{\prime }u_{j}^{\prime }}$$

(15) $$-\rho \bar{u_{i}^{\prime }u_{j}^{\prime }}=\mu_{t}\left(\frac{\partial u_{i}}{\partial x_{j}}+\frac{\partial u_{j}}{\partial x_{i}}\right)-\frac{2}{3}\rho k\delta_{\mathrm{ij}}$$

In the above equations *μ* is the dynamic viscosity, $\mu_{t}$ is the eddy viscosity, *ρ* is the fluid density, *k* is the turbulent kinetic energy, $\delta_{\mathrm{ij}}$ is the Kronecker delta, $u_{i}$ is the velocity component and $u_{i}$ is the spatial component.

## 3 Results

### 3.1 Variation of blades’ deformation and pressure rise during FSI simulation iterations

**Figure 5:**
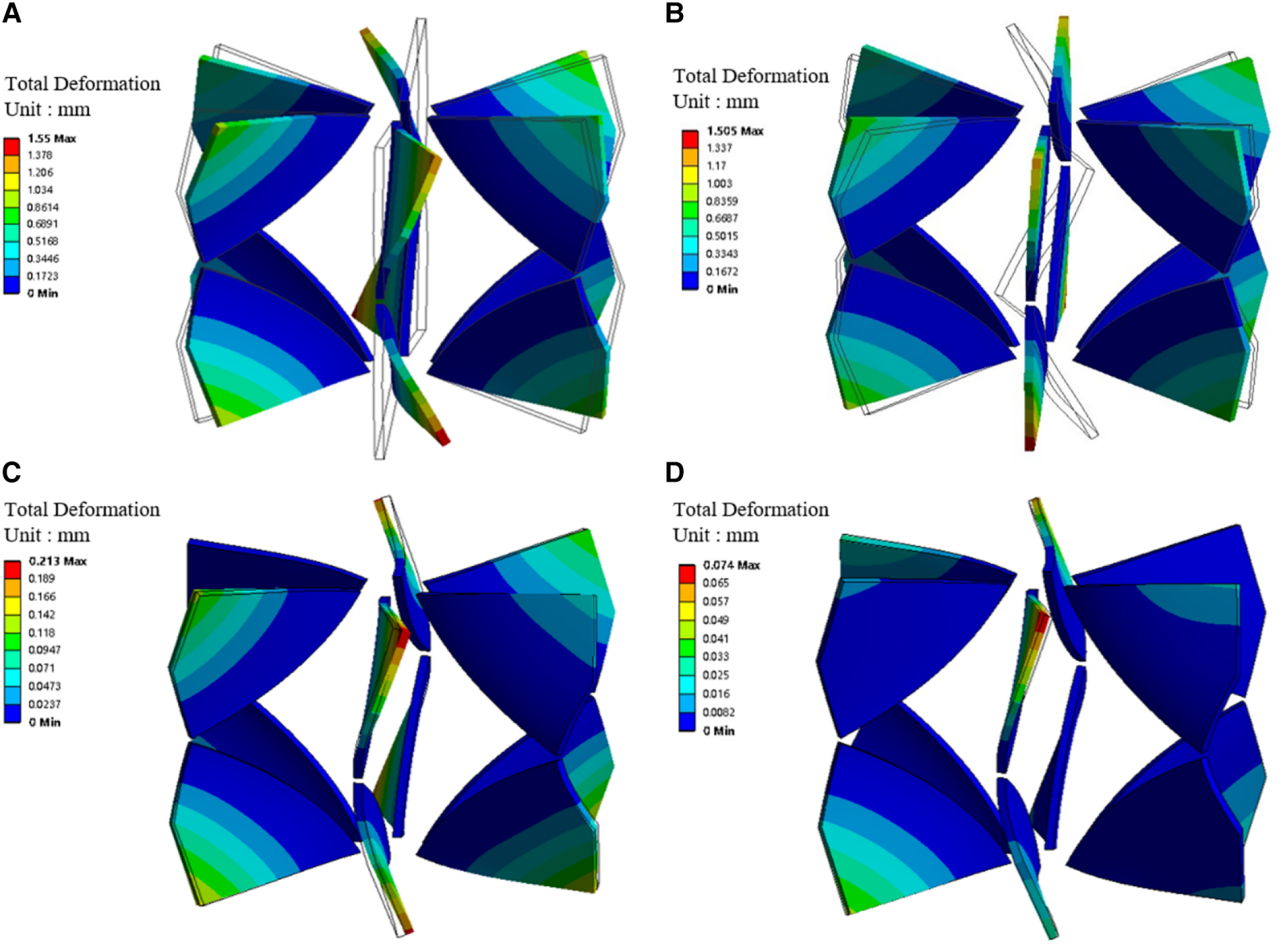
Blades' deformation during the one-way FSI simulation iterations. (**A**) Blades' deformation obtained from the first round of FSI; (**B**) Blades' deformation under stress; (**C**) Blade deformation obtained from the second round of FSI; (**D**) Blade deformation obtained from the third round of FSI. (Dotted frame represents the blade shape before deformation, solid body represents the blade shape after deformation).

Figure 5; Table 1 illustrate the changes in blades’ deformation and blood pressure rise during FSI simulation iterations. Figures 5A,C,D sequentially show the blades’ deformation during three rounds of FSI simulation. It is evident that as the iterations progress, the solid body gradually aligns with the dashed frame. Additionally, the maximum deformation of the blades gradually decreases, indicating that the blade shape is stabilizing. According to Table 1, the pressure rises in both the SVC and IVC also tend to stabilize during the iteration process. The pressure rises of the last two iterations differing by less than 5%. These results validate that the one-way FSI simulation used in this paper can achieve a stable blade shape and the corresponding pressure rise.

**Table 1:** Maximum blade deformation and blade pressure rise during three rounds of one-way FSI simulation.

	First round	Second round	Third round
Maximum blade deformation(mm)	1.551	0.213	0.074
DP-SVC (Pa)	439.14	465.94	443.36
DP-IVC (Pa)	313.45	345.36	350.05

DP-SVC indicates the blood pressure rise in the SVC, calculated as the pressure at the PA outlet minus the pressure at the SVC inlet. DP-IVC indicates the blood pressure rise in the IVC, calculated as the pressure at the PA outlet minus the pressure at the IVC inlet.

Moreover, stress exists within the blade in the second and subsequent rounds of FSI simulation. Figures 5A,B were compared with each other to verify the accuracy of applied stress. Figure 5A shows the blade bending under surface pressure. Figure 5B depicts the blade returning to vertical shape after the removal of surface pressure, while stress is retained. The maximum deformations in Figures 5A,B are 1.55 mm and 1.505 mm, respectively. The difference is only 3%, indicating that the stress is sufficiently precise.

### 3.2 Blood pressure rise corresponding to blades with different elastic moduli at two rotational speeds

**Figure 6:**
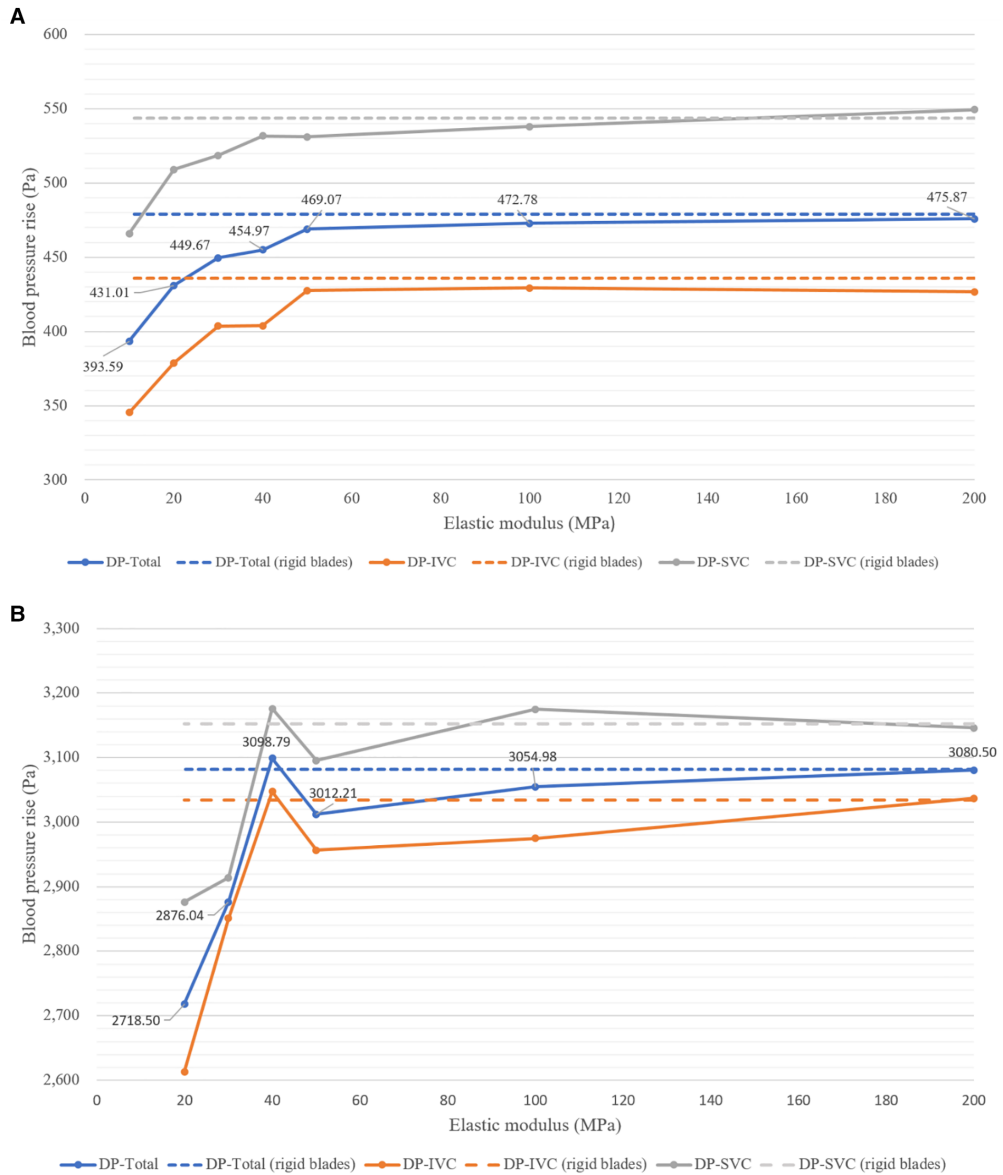
Blood pressure rise corresponding to blades with different elastic moduli at two rotational speeds. (**A**) At 4,000 rpm; (**B**) at 8,000 rpm.

Figure 6 displays the blood pressure rise corresponding to flexible blades and rigid blades. DP-Total represents the total pressure rise, obtained by the weighted sum of DP-SVC and DP-IVC, based on the flow rates in SVC and IVC (DP-Total = DP-SVC × 0.4 + DP-IVC × 0.6). At a speed of 8,000 rpm, blades with an elastic modulus of 10 MPa deform excessively and lose their functionality entirely. Consequently, data related to this condition are not included in the analysis.

From Figure 6A, it can be observed that at 4,000 rpm, the pressure rises corresponding to flexible blades show an increasing trend. As the elastic modulus increases, DP-SVC, DP-IVC and DP-Total all gradually increase. The pressure rises corresponding to flexible blades with elastic moduli of 50 MPa, 100 MPa, and 200 MPa are relatively close to those of rigid blades. Figure 6B reveals that at 8,000 rpm, the pressure rises corresponding to flexible blades also trends upward. However, when the elastic modulus of the blades increases from 40 MPa to 50 MPa, the pressure rise decreases instead. The pressure rises corresponding to flexible blades with elastic moduli of 40 MPa, 50 MPa, 100 MPa, and 200 MPa are relatively close to those of rigid blades. Notably, the pressure rise corresponding to the 40 MPa elastic modulus flexible blade is higher than that of the rigid blades. Comprehensive analysis indicates that in most cases, blade deformation leads to a decrease in functionality. However, at the highest rotational speed, there are instances where blade deformation results in an increase in functionality.

### 3.3 Hemolysis risk corresponding to blades with different elastic moduli at two rotational speeds

**Figure 7:**
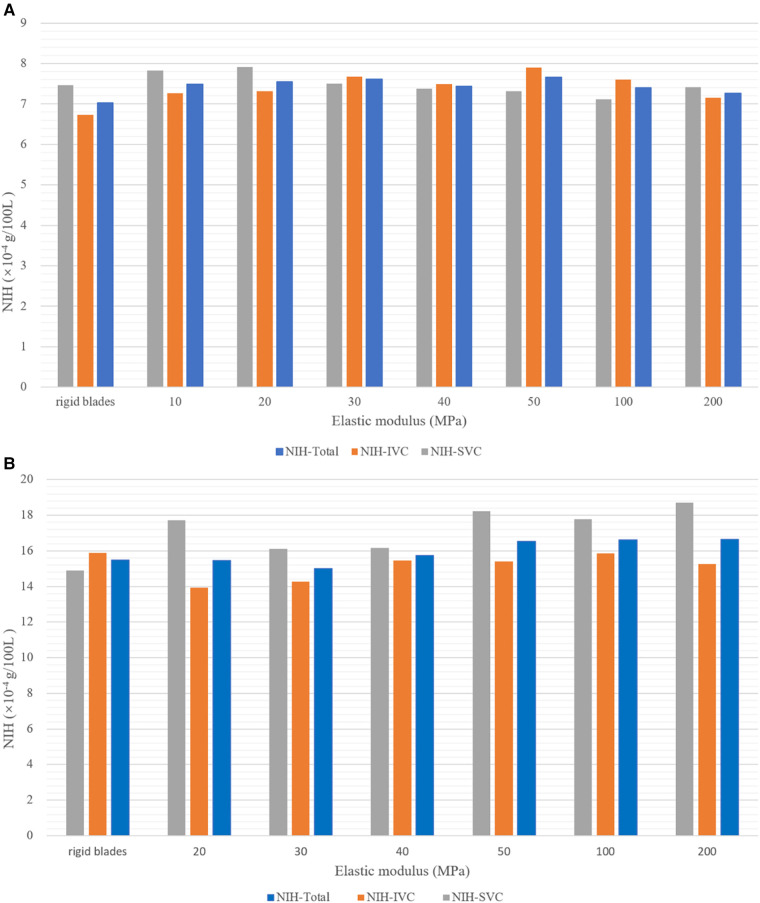
Hemolysis risk corresponding to blades with different elastic moduli at two rotational speeds. (**A**) At 4,000 rpm; (**B**) at 8,000 rpm.

Figure 7 shows the hemolysis risk associated with flexible blades and rigid blades. After completing the streamline number independence test, 1,100 streamlines were released at both the SVC and IVC inlets. The NIH values for each streamline were calculated and average values were used to represent the hemolysis risk in the blood flow of the SVC and IVC (NIH-SVC and NIH-IVC). The overall hemolysis risk (NIH-Total) was derived by the weighted sum of NIH-SVC and NIH-IVC (NIH-Total = NIH-SVC × 0.4 + NIH-IVC × 0.6). The results indicate that at both rotational speeds, the hemolysis risks associated with flexible and rigid blades are relatively similar. At 4,000 rpm, the NIH-Total for flexible blades ranges between 7.26 × 10^−4^ and 7.67 × 10^−4^ g/100 L, with a maximum difference of 5.6%. The NIH-Total for rigid blades is 7.03 × 10^−4^ g/100 L. Therefore, the difference in NIH-Total between flexible and rigid blades ranges from 3.3% to 9.1%. At 8,000 rpm, the NIH-Total for flexible blades ranges between 15.01 × 10^−4^ and 16.65 × 10^−4^ g/100 L, with a maximum difference of 10.9%. The NIH-Total for rigid blades is 15.48 × 10^−4^ g/100 L.As a result, the difference in NIH-Total between flexible and rigid blades ranges from 0.2% to 7.6%.

Under the same rotational speed, flexible blades with different elastic moduli have varying shapes, yet the corresponding hemolysis risks are similar. This suggests that the impact of rotational speed on hemolysis risk is greater than that of blade deformation. Moreover, literature data indicate that the NIH values for HeartMateII (Thoratec Inc., Pleasanto, California, USA) and CH-VAD (CH Biomedical, Inc., Suzhou, China) at medium speeds are 5.83 × 10^−3^ g/100 L and 1.35 × 10^−3^ g/100 L, respectively (28). The highest NIH-Total in this study is 1.665 × 10^−3^ g/100 L, falling between the NIH values of HeartMateII and CH-VAD. This result demonstrates that the assist device proposed in this paper is reliable in terms of hemolysis.

### 3.4 Thrombus risk corresponding to blades with different elastic moduli at two rotational speeds

**Figure 8:**
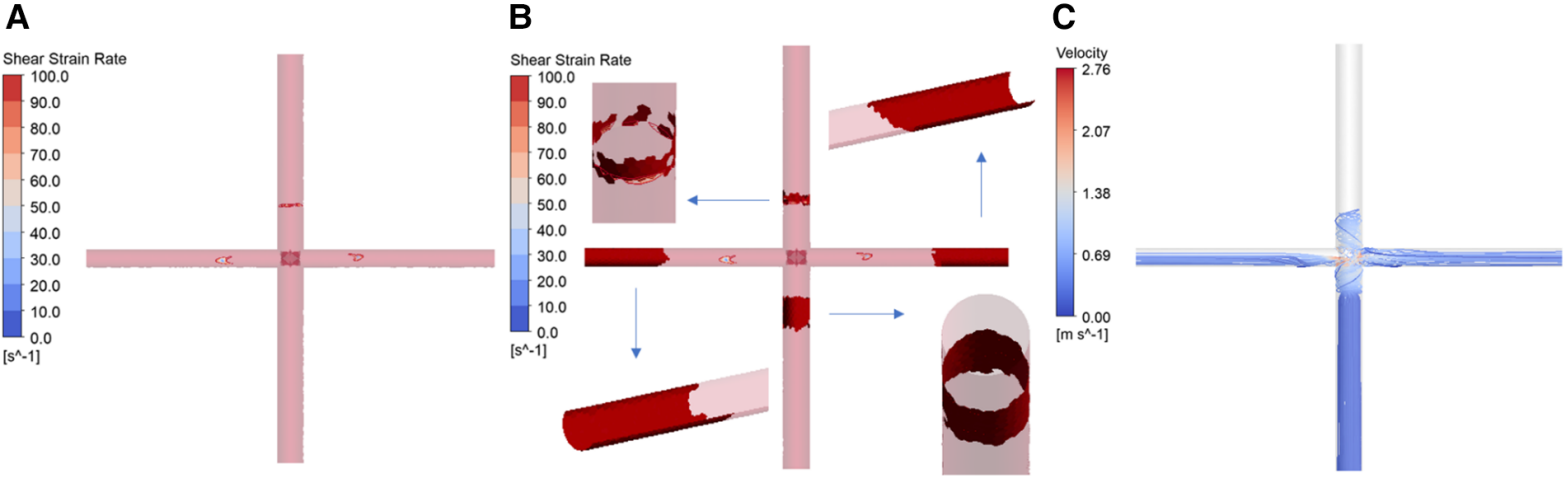
Simulation results of thrombus formation. (Corresponding to rotational speed of 4,000 rpm, blade elastic modulus of 10 MPa. (**A**) Distribution of wall shear strain rate; (**B**) thrombus distribution; (**C**) IVC streamline diagram).

Figure 8 presents the results of thrombus formation simulation, corresponding to a rotational speed of 4,000 rpm and a blade elastic modulus of 10 MPa. As mentioned earlier, the shear strain rate (SSR) is utilized to determine areas prone to thrombus growth. The concentration of AP is used for quantitatively assessing the thrombus risk. To enhance readability, the AP concentration is scaled and represented as the SAP index. This index has a minimum value of 0, corresponding to the initial AP concentration (25 × 10^12 ^/m³). Each unit increase indicates an increase of one millionth of the initial AP concentration. Figure 8A shows the distribution of wall SSR. It is observed that regions with SSR less than 100 /s are only present on the walls of the SVC, LPA and RPA. There are no low SSR areas on the blades’ surfaces. Figure 8B, based on Figure 8A, displays grids where the SAP exceeds 23 in a dark red color. Detailed examination through close-up and cross-sectional views reveals that all four vessels exhibit wall grids with higher SAP. However, IVC lacks regions with low SSR, and the high SAP at the LPA and RPA outlets is due to the transport of AP. Only the wall of the SVC displays areas with both low SSR and high SAP, indicating that thrombus is more likely to form on the SVC wall. Figure 8C shows streamlines released from the IVC. Due to the larger flow volume in the IVC, a portion of the blood enters the SVC, leading to a collision within the SVC. This collision area has a low SSR, which facilitates the deposition of AP and thus the formation of thrombus. At both rotational speeds, the thrombus formation scenarios for different blades are similar to those depicted in Figure 8B. Thrombus are more likely to growth on the wall of SVC, hence separate illustrations are not provided.

A statistical analysis of the mean SAP values in the SVC for different blades provides the following insights: At 4,000 rpm, the mean SAP value for flexible blades with various elastic moduli ranges between 2.60 and 2.68, with a maximum difference of 3%. The mean SAP value for rigid blades is 2.60. As a result, the maximum difference in the thrombus risk between flexible and rigid blades is 3%. At 8,000 rpm, the mean SAP value for flexible blades with different elastic moduli ranges from 14.92 to 15.88, with a maximum difference of 6%. The mean SAP value for rigid blades is 15.24. Therefore, the maximum difference in thrombus risk between flexible and rigid blades is 4.2%. These results indicate that, in terms of thrombus formation, the impact of rotational speed is also much greater than that of blade deformation.

## 4 Discussion

The Fontan surgery successfully diverts venous blood into the lungs by constructing the TCPC structure. The issue of mixing between arterial and venous blood has been resolved. However, long-term reliance on a single ventricle to power both systemic and pulmonary circulations can lead to various complications, such as elevated central venous pressure, increased pre-load on the single ventricle, and insufficient pulmonary perfusion. Patients are at high risk of developing single ventricle heart failure. Numerous scholars have proposed a variety of solutions to provide additional power for the Fontan circulation. Based on the location of the assistive device, these solutions can be categorized into extracorporeal and intracorporeal assistance. Trusty et al. (29) used pulsatile blood pumps, conduits, and baffles to explore efficient extracorporeal assistance methods in an *in vitro* loop. Their findings revealed that without partitions, blood pumped out of the pump's outlet tube could be reabsorbed by the inlet tube, causing recirculation. Effective power assistance was only possible by completely separating the inlet and outlet tubes. Wang et al. (8, 9) used dual-lumen catheters and extracorporeal blood pumps for Fontan support. The successful assistance achieved was also due to the use of valves or umbrella-like membranes that separated the inlet and outlet of the dual-lumen catheters. Therefore, providing extracorporeal assistance to patients requires the addition of specific obstructions to the existing TCPC structure, increasing the number of devices and the complexity of surgery. Intracorporeal assistance methods are more convenient as they do not alter the existing TCPC structure. Among these, collapsible assist devices have a smaller size during implantation, leading to reduced invasiveness. After implantation, these devices expand, increasing in diameter. Compared to rigid devices of similar implantation size, collapsible devices operate at lower speeds to achieve the same pressure rise. Therefore, the temperature increase, mechanical wear, and blood damage during the operation of collapsible pumps are all lower (30).

In the field of ventricular assist devices, the folding mechanisms of flexible blades mainly include mechanical folding, material folding, and hybrid folding. Mechanical folding typically employs hinged structures (31–33), requiring locking components to ensure the blades’ operational shape. However, the use of complex blood-immersed joint significantly increases thrombus risks. Material folding relies on the material’s properties for folding, offering simpler structures (34–36). This approach requires the blades to exhibit two distinct states: one for elastic deformation into the stored position, and another to resist hydrodynamic forces during operation. The hybrid folding mechanism combines the advantages of the previous two methods. It involves designing unique structures for flexible blades to achieve structural anisotropy (37, 38). This mechanism allows the device to be both easily collapsible and capable of maintaining its operational shape. In the latter two folding mechanisms, the blade deformation and its impact on device performance are particularly crucial for product development. FSI simulation can rapidly explore relevant patterns, offering guidance for material selection and structural design.

For high-speed rotating devices, conducting two-way FSI simulation can yield results that are closer to reality. However, this method requires a significant amount of resources. At the early stage of research, employing one-way FSI simulation can also reveal certain patterns while substantially reducing resource consumption. The results demonstrate that the one-way FSI simulation described in this paper can accurately model the stable shape of flexible blades. This allows for the investigation of the impact of blade deformation on device performance, aiding in the determination of the optimal blade elastic modulus. It is important to note that one-way FSI simulation neglects the impact of blade deformation velocities and accelerations on the fluid, failing to capture transient flow fluctuations. This leads to some deviation in the calculations of blood pressure rise, hemolysis risk, and thrombus risk. However, these neglected fluctuations are primarily near the impeller, and their impact area is limited. As presented in the paper, blood pressure rise is largely determined by the rotational speed and blade morphology. Furthermore, the hemolysis risk and thrombus risk are mainly influenced by the rotational speed. Compared to these factors, the impact of flow fluctuations is relatively minor. Although one-way FSI simulation overlooks the influence of structural dynamics on the fluid, it still accurately depicts the impact of the main factors on performance indicators. This method can be further applied to the entire impeller to explore the effect of hub deformation. For flexible left ventricular assist device, such as impella ECP (Abiomed, Danvers, MA), one-way FSI simulation is equally applicable.

This study has the following limitations: (1) It did not analyze the entire device, thus ignoring the impact of components other than the impeller on blood pressure rise, hemolysis risk, and thrombus risk; (2) Fluctuations in rotational speed caused by the operation of the brushless motor were not considered; (3) The one-way FSI simulation can't capture blades’ status at each moment; (4) No experiments were conducted to verify the accuracy of the simulation results; (5) This study employs steady-state boundary conditions and an ideal TCPC model for research, neglecting the effects of blood pulsatility and real vascular geometry. The future research plans include: (1) Modeling and simulating the entire device; (2) Constructing an in-vitro experimental system, using a high-speed camera to capture the shape of flexible blades during rotation, and comparing it with the simulation results; (3) Conducting hydraulic experiments, particle image velocimetry (PIV) experiments, hemolysis experiments, and thrombosis experiments to verify the simulation results of pressure rise, velocity field, hemolysis risk, and thrombus risk; (4) Collecting data on rotational speeds during device operation and applying these varying speeds in FLUENT using a user-defined function (UDF); (5) Exploring two-way FSI simulation methods and using transient simulation to study the impact of blade flutter on the flow field; (6) Performing patient-specific studies using measured boundary conditions and real TCPC models.

## 5 Conclusion

This article proposed a right heart assist device equipped with flexible blades. A one-way FSI simulation was used to study the deformation of the flexible blades and its impact on the device's performance. The simulation results indicate that within the studied range (rotational speeds of 4,000 rpm and 8,000 rpm, elastic modulus between 10 MPa and 200 MPa), deformation of the flexible blades leads to a decrease in functionality. However, within a certain range of elastic modulus, the pressure rise associated with flexible blades is close to that of rigid blades. Additionally, compared to blade deformation, rotational speed has a more significant impact on the hemolysis risk and thrombus risk. After a comprehensive analysis of blade compressibility, blood pressure rise, hemolysis risk, and thrombus risk, the optimal elastic modulus for the flexible blades is determined to be between 40 MPa and 50 MPa.

## Data Availability

The original contributions presented in the study are included in the article/**Supplementary Material**, further inquiries can be directed to the corresponding author.
