# Supplementary material for: Study on the optimal elastic modulus of flexible blades for right heart assist device supporting patients with single-ventricle physiologies
Source: Front Cardiovasc Med. 2024 Mar 25;11:1377765. doi: 10.3389/fcvm.2024.1377765 (PMC10999545; doi:10.3389/fcvm.2024.1377765)
Supplement: Supplementary file 1 [file Datasheet1.pdf]

# *Supplementary Material*

In this supplementary document, a CFD simulation was conducted on the centrifugal blood pump provided by the FDA for six different operating conditions [1]. Based on the obtained flow field information, Equations 5 and 6 in the main text were used to assess the hemolysis risk for each condition. These simulation results, combined with experimental data, facilitated the determination and verification of parameter B. The specific process included the following steps: Firstly, the simulated flow field data for condition 5 were compared with the measured data to validate the accuracy of the simulation methods (as the literature only provided flow field data for condition 5 [1]). Secondly, the relationship between the simulation NIH value for condition 5 and parameter B was established. Then, the value of parameter B was determined using the experimental NIH value of condition 5. Finally, the determined value of parameter B was used to calculate the hemolysis risk for the remaining conditions. These calculations were then compared with experimental hemolysis risk results to verify the reliability of the chosen value.

The assembly diagram, detailed view, computational model, and extracted fluid domain of the centrifugal blood pump are shown in Supplementary Figure 1 [2]. The six operating conditions involved in this analysis are presented in Supplementary Table 1. In CFD simulation, the velocity distribution at the pump inlet was set according to the measured results. Supplementary Figures 2A and 2B illustrate the measured data and simulation setting of inlet velocity for an example flow rate of 6 L/min [2]. The pump outlet was set as a zero-pressure boundary condition. Blood was modeled as a Newtonian fluid with a density of 1060 kg/m$^3$ and a dynamic viscosity of 3.5 cP. Additionally, the SST k-ω model was used in the simulation as the Reynolds numbers for all operating conditions were far greater than 4000. The fluid domain was meshed as a polyhedral grid using Fluent Meshing 2020R2 (Canonsburg, USA). After a grid independence study, the fluid mesh consisting of approximately 15.3 million elements was used for simulation.

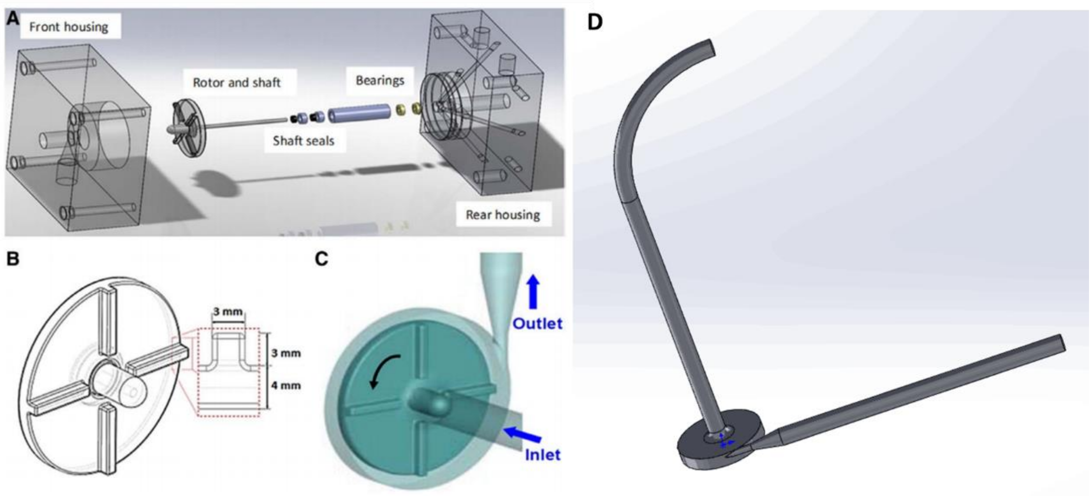


**Supplementary Figure 1.** FDA blood pump model

(A) Assembly diagram; (B) Enlargement of rotor; (C) Computational model; (D) Flow domain

**Supplementary Table 1**. Six operational conditions analyzed in the study

| Condition | Flow Rate (L/min) | Pump Speed (rpm) | Reynolds Number |
|---|---|---|---|
| 1 | 2.5 | 2500 | 209338 |
| 2 | 2.5 | 3500 | 293073 |
| 3 | 4.5 | 3500 | 293073 |
| 4 | 6.0 | 2500 | 209338 |
| 5 | 6.0 | 3500 | 290373 |
| 6 | 7.0 | 3500 | 293073 |

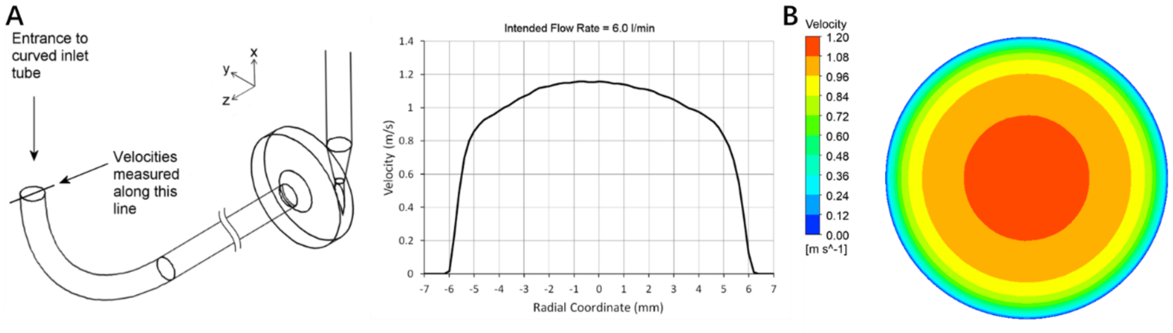


**Supplementary Figure 2.** Measured data and simulation settings of inlet velocity

(Corresponding to a flow rate of 6 L/min. (A) Measured data; (B) Simulation settings)

Supplementary Figure 3 presents the measured and simulated results of two-dimensional velocities on a cross-section under condition 5 [1]. This cross-section is 1.2mm from the top of the impeller, aligning with the mid-axial plane of the diffuser. Comparing Supplementary Figures 3A and 3B, it is evident that the simulation results closely match the measured data. In the blade-passage, as the radial distance increases, the velocity initially increases and then decreases, with the maximum velocity values occurring near the edges of the blades. Furthermore, the jets in the diffuser area are all skewed towards the outer walls. Supplementary Figure 4 illustrates the variation of two-dimensional velocities along two paths within the cross-section [1]. The comparison between Supplementary Figures 4A1 and 4A2 shows that the CFD simulation accurately captures the trend and absolute values of velocity along the radial path. Observing Supplementary Figures 4B1 and 4B2, it is evident that the CFD simulation also accurately captures the trend of velocity along the horizontal path, although there is a certain deviation in the peak velocity values compared to the experimental data. These results validate that the CFD simulation has obtained an accurate flow field, which can be further utilized for identifying parameter B.

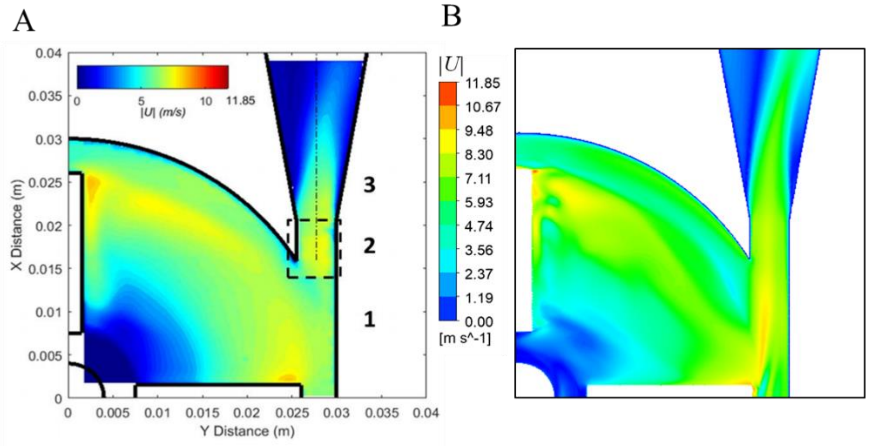


**Supplementary Figure 3.** Distribution of two-dimensional velocity on upper-blade plane under condition 5

(A) Experimental results; (B) Simulation results; 1, 2, and 3 represent the blade-passage, cutwater, and diffuser regions, respectively.

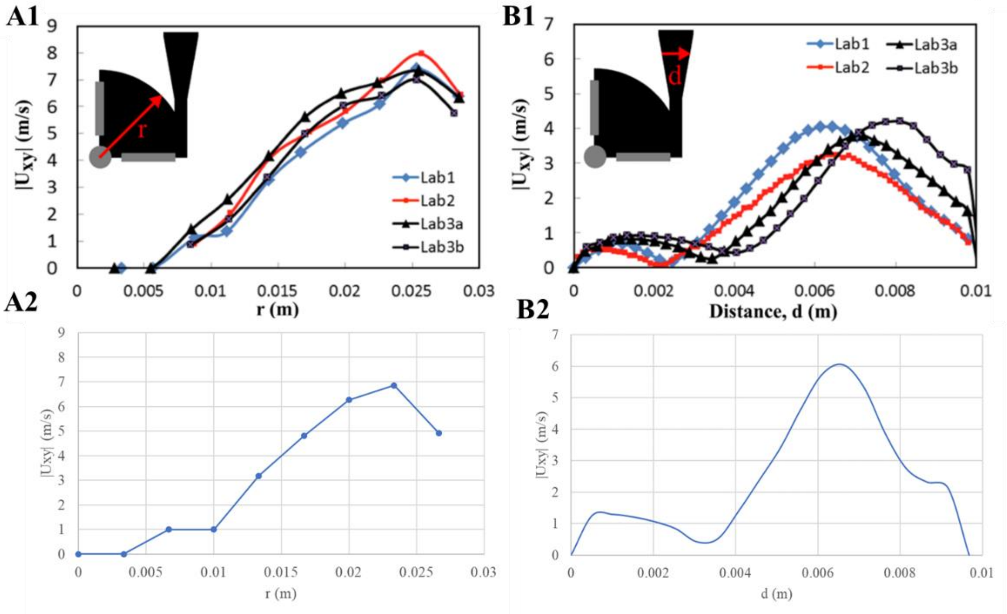


**Supplementary Figure 4.** Variation of two-dimensional velocity along radial and horizontal paths under condition 5

(A1) (B1) Experimental results obtained from three laboratories. (A2) (B2) Simulation results of this study

The calculation method for obtaining the NIH value based on hemolysis experimental data is shown in equation 1 [3].

$$NIH_{exp}(g/100L) = \frac{\Delta fHb \times V \times [1 - \frac{Ht}{100}] \times 100}{\Delta t \times Q} \quad \textbf{(1)}$$

In this equation, $\Delta fHb$ represents the measured increase in plasma-free hemoglobin ($Hb$) during the test period (g/L), $V$ is the blood volume in the test circuit (L), $Ht$ denotes the hematocrit (%), $\Delta t$ is the duration of the test (minutes), and $Q$ signifies the flow rate (L/min). For condition 5, the measured data of the above parameters are as follows: $\Delta fHb = 0.41\text{g/L}$，$V = 0.24\text{L}$，$Ht = 36\%$, $\Delta t = 120\ \text{min}, Q = 6\text{L/min}$ [2]. Based on these data, the $NIH_{exp}$ corresponding to condition 5 was calculated to be 0.008747 g/100L.

The simulation NIH value was calculated according to Equations 5 and 6 in the main text. After completing the streamline number independence test, 2000 streamlines were released at the pump inlet. The NIH values for each streamline were calculated, and the average value was used to represent the overall hemolysis risk. Supplementary Figure 5 illustrates the relationship between the simulation NIH value and parameter B, corresponding to condition 5. It is observed that when B is set to 0.55, the simulation NIH value is 0.008816 g/100L, differing only by 0.79% from the experimental NIH value. Furthermore, the simulation NIH values for the remaining operating conditions were also calculated, with B set to 0.55. The relative hemolysis index (RHI) for each operating condition was obtained by normalizing their respective simulation NIH values relative to the simulation NIH value of condition 5, as shown in Supplementary Figure 6 [1]. It is indicated that for conditions 1-4, the RHIs obtained in this study closely match the experimental results. For condition 6, however, the RHI obtained shows some differences from the experimental data, yet it aligns with the calculation results of most other research groups. Above comparison proves that selecting a value of 0.55 for B allows for a relatively accurate assessment of the hemolysis risk in centrifugal blood pumps or rotary devices with similar operating principles.

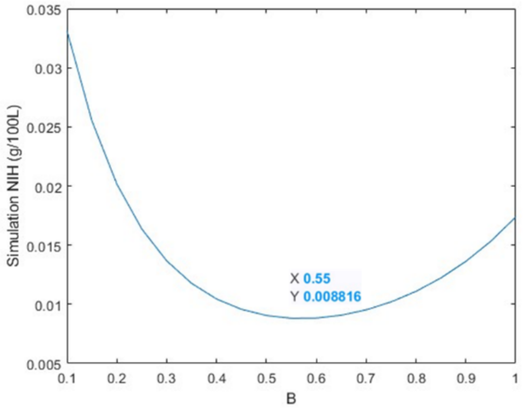


**Supplementary Figure 5.** Relationship between simulation NIH and parameter B

(Corresponding to operating condition5)

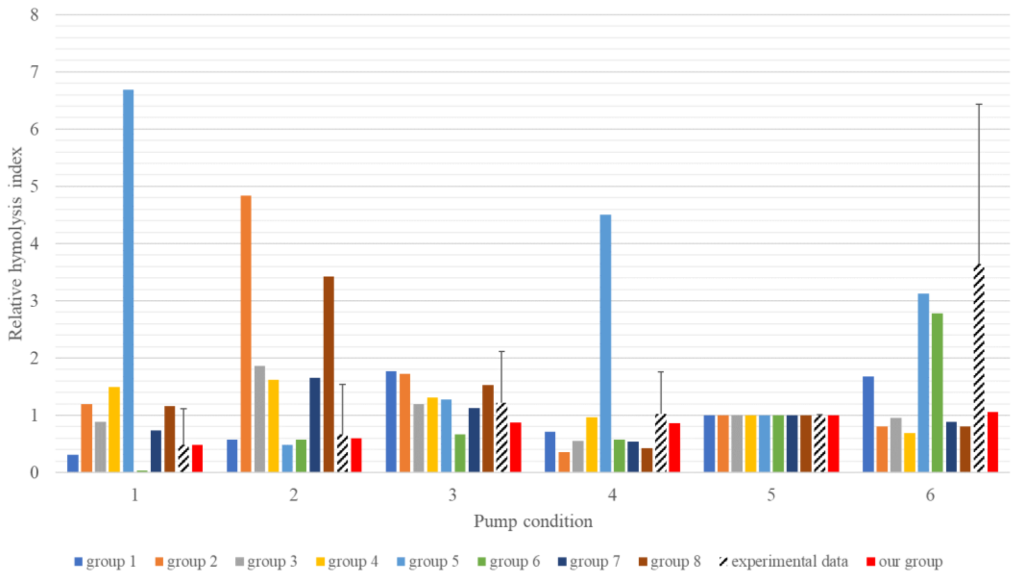


**Supplementary Figure 6.** Relative hemolysis index for each operating condition

(Data of groups 1 to 8 represent the RHI obtained through simulations by various research groups. Experimental data are shown in the form of mean values with standard deviations. The RHI obtained in this study is indicated in red.)

## Supplementary Reference


1. Malinauskas RA, Hariharan P, Day SW, Herbertson LH, Buesen M, Steinseifer U, et al. FDA benchmark medical device flow models for CFD validation. Asaio J. (2017) 63:150-160. doi:10.1097/MAT.0000000000000499

2. U.S. Food and Drug Administration. (2016). Computational Fluid Dynamics: An FDA Critical Path Initiative. https://fdacfd.nci.nih.gov [Accessed 25 Jan, 2024].

3. Koller T, Hawrylenko A. Contribution to the in vitro testing of pumps for extracorporeal circulation. *J Thorac Cardiovasc Surg.* (1967) 54:22–29. doi:10.1016/S0022-5223(19)43109-7
